# Supplementary material for: Are Tumor Marker Tests Applied Appropriately in Clinical Practice? A Healthcare Claims Data Analysis
Source: Diagnostics (Basel). 2023 Nov 3;13(21):3379. doi: 10.3390/diagnostics13213379 (PMC10648915; doi:10.3390/diagnostics13213379)
Supplement: Supplementary file 1 [file diagnostics-13-03379-s001.zip › diagnostics-2672673-supplementary.pdf]

diagnostics

MDPI

Supplementary Material

Table S1. Tumor marker (TM), indications for determination and cancer related diseases and interventions (CDI).

TM	Indications for TM determination according to medical guidelines	Antineo-plastic (ATC[a])	Diagnoses (ICD-10[b])	Inpatient operation (CHOP[c])	Outpatient operations and procedures (TARMED[d])
**All TM**		L01			37.0210-0270 (histology), 39.4060 and 39.4080 (CT-thorax/abdo men), 32 (radio oncol-ogy), 00.1530 (>2 visits to oncologist)
**CEA**	According to international guidelines, CEA should be determined before operating on **colorectal cancer** and afterwards every three to six months (depending on guideline) for two years and every six to 12 months for the following 3 years [10]. It can be used in ad-vanced colorectal cancer for therapy monitoring as well. It should not be used as a screening parameter [20]. In the rare cases of medullary thyroid cancer, CEA can be (in addition to Calcitonin) a “valuable diagnostic, prognostic and predictive marker” ac-cording to guidelines, since its serum concentration is related to the tumor cell mass [18,23,36]. However, it should not be used with diagnosis of every thyroid nodule [18]. Because of the latter, thyroid cancer in ICD-10 was not coded as appropriate for CEA de-termination.	L01	C18, C19, C20 (colorectal cancer)	45.7, 45.8, 48.3511, 48.5, 48.6	20.1540, 20.1550, 20.1570, 20.1580, 20.1590, 20.1600, 20.1610
**NSE**	NSE should be determined at least once with diagnosis of a **neuroendocrine carcino-ma** [6,22]. It should like other TM not be used for cancer screening. At least since the year 2000 it is not recommended for **lung cancer** diagnosis and follow-up in German speaking guidelines [37,38]. There is no routine indication according to current guidelines for other tumor entities like Merkel carcinoma, neuroblastoma or metastasized melanoma [39], but it is used in medical praxis for the previous indications.	L01	C34 (lung cancer)	for lung cancer: 32.2, 32.4, 32.5, 32.6	for lung cancer: 16.0620, 16.0630, 16.0640, 16.0650, 16.0660, 16.0670, 16.0690, 16.0700
**Calcitonin**	For the diagnosis of **medullary thyroid cancer** (MTC), demonstration of calcitonin ex-pression is mandatory [18,35]. Calcitonin is a prognostic and predictive marker in MTC. However, it is not recommended by guidelines to determine Calcitonin in every patient with thyroid nodules [35,36,40].	L01	C73 (thyroid cancer)	06.4, 06.5, 06.2, 06.3	14.0170, 14.0180, 14.0190, 14.0200, 14.0210, 14.0220
**CA 15-3**	According to guidelines, the determination of CA15-3 is part of the work-up in metasta-sized **breast cancer** and should neither be used in diagnosis nor in follow- up of breast cancer patients, in so far there are no clinical abnormalitie [12,14] .	L01, L02B	C50, D05 (breast can-cer)	85.A0-A7	23.0110- 23.0210
**CA 19-9**	With diagnosis of a **pancreatic mass**, CA19-9 should be determined [8,31]. while there is no recommendation for screening [32] or a standardized follow-up (including TM) after curatively resected pancreatic cancer. Additionally, it can be determined for the diagnosis and follow-up for **biliary tract carcinoma**, as well as in screening for biliary cancer in patients with primary sclerosing cholangitis [54].	L01	C25 (pan-creas), C22.1,7,9, C23, C24 (biliary)	52.5 to 52.63; 51.2, 51.3, 51.99, 50.20-29, 50.2A2-50.2C16	20.2710 - 20.2750 20.2610-20.2700; 20.2470-20.2530
**CA 72-4**	At least since the year 2012 there is no guideline based recommendation for the use of this (or other, e.g. CEA, CA19-9) markers in **gastric cancer** [9,21]. A routine determination of TM should, according to guidelines, not be performed either in primary diagnosis nor in follow-up. It is used in medical praxis for gastric cancer progress control.	L01	C16 (gastric cancer)	43.42.0, 43.5, 43.6, 43.7, 43.8, 43.9	20.1050, 20.1100, 20.1110, 20.1120, 20.1130, 20.1140

**CA 125**	CA125 should not be used as a screening parameter, even not in high risk patients of **ovarian cancer**: In a retrospective study of 241 women with a pathogenic BRCA1 or BRCA2 mutation the positive predictive value of a determination of CA125 was only six % [33]. In patients with ovarian cancer there should not be a routine follow-up with determination of markers insofar the patient is clinically symptom free [7,34]. This is different in patients with germ cell tumor: here it is recommended to determine the marker every month in the first six months after diagnosis. CA125 should be determined under maintenance therapy in both entities.	L01	C56	65.3, 65.4	22.1570, 22.1580, 22.1590

[a] Anatomical therapeutic chemical classification [55] [b] International Statistical Classification of Diseases and Related Health Problems [56] [c] Swiss operation classification.[57] [d] "Tarif médical" (TARMED), the Swiss single service tariff for medical services [58].

Table S2. Number of tumor marker (TM) determination in one patient at index date.

Characteristic	1 TM, N = 25,728[a]	2 different TM, N = 7838[a]	3 or more different TM, N = 2971[a]
**Gender**			
Women	17,075 (66.4%)	5492 (70.1%)	2603 (87.6%)
Men	8653 (33.6%)	2346 (29.9%)	368 (12.4%)
**Age**	66 (54, 76)	68 (56, 76)	65 (54, 75)
**Appropriate request[b]**	10.038 (39.0%)	3985 (50.8%)	1150 (38.79%)

[a] Number (percent); Median (IQR). [b] Percent of "Appropriate request" is given in relation to the respective number of TM determined.

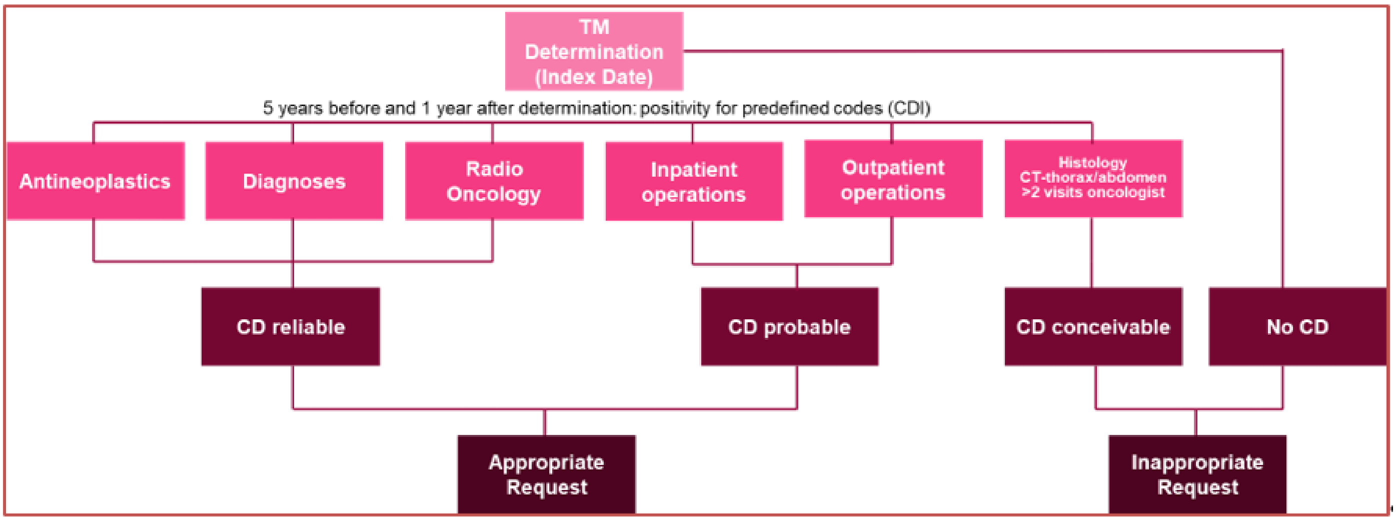

Figure S1. Flow Chart: Tumor marker (TM) determination, cancer related diseases and interventions (CDI), and classifica-tion towards appropriateness according to the probability of cancer diagnosis (CD).
